# Relationship between estrus endometrial edema and progesterone production in pregnant mares two weeks after ovulation

**DOI:** 10.1186/s12917-022-03512-0

**Published:** 2022-11-21

**Authors:** Anna Grabowska, Roland Kozdrowski

**Affiliations:** 1 Anawet Sp. Z O.O., Ul. Spółdzielcza 3, 62-800 Kalisz, Poland; 2 Faculty of Biological and Veterinary Sciences, Nicolaus Copernicus University, Toruń, Poland

**Keywords:** Mare, Endometrial edema, Progesterone, Early pregnancy

## Abstract

**Background:**

Progesterone plays a crucial role in the maintenance of pregnancy from conception to about 100–120 days of gestation when placenta becomes the main source of gestagens. The aim of the study was to test progesterone concentration 14 days after ovulation in pregnant mares and relate it to peak estral endometrial edema and the presence of intrauterine fluid (IUF) after artificial insemination (AI), the number of treatments against IUF, and the time from AI to the day when the uterus was found free of fluid.

**Results:**

Mares were divided into two groups: group A (*n* = 13; age 10.8 ± 4.5 years) in which a normal embryonic vesicle with a diameter ≥ 14 mm and a corpus luteum with a diameter ≥ 15 mm were found 14 days after ovulation, and group B (*n* = 22; age 9.4 ± 4 .0 years) in which 14 days after ovulation, a small (< 15 mm) corpus luteum and/or a small embryonic vesicle was observed (diameter < 14 mm). Mares from group A had a significantly higher progesterone concentrations at 14 days after ovulation compared with group B mares. The presence of IUF, the number of treatments against IUF, and the time from AI to the day when uterus was found free of fluid did not affect progesterone concentration measured 14 days after ovulation. In group B, a significant correlation was found between progesterone concentration measured 14 days after ovulation and endometrial edema evaluated during estrus.

**Conclusions:**

In some cases poor development of endometrial edema during estrus can be associated with lower progesterone production 14 days after ovulation. Nevertheless, scientific explanation for this finding cannot be given based on our study.

## Introduction

The incidence of early embryonic death (EED) reported in the literature ranges from 2.6% to 24%, and in mares older than 18 years EED incidence can exceed 30% [1]. Reduction in reproductive efficacy due to EED is a serious veterinary and breeding problem directly related to financial losses.

Undoubtedly, EED has a multifactorial background. Factors responsible for EED can be divided into intrinsic, extrinsic, and embryonic [1]. Intrinsic factors, including age of the mare and endometrial diseases, seem to play the most important role in the pathogenesis of EED. Ageing of the mare is strictly associated with decreasing oocyte quality [2]. Additionally, older mares are more susceptible to endometritis, and the development of endometrosis is also age dependent [3]. However, the dysfunction of primary corpus luteum and, consequently, the decrease in progesterone concentration during early pregnancy can still be considered a hypothesis rather than a fully documented intrinsic cause of EED [1, 4, 5].

Progesterone plays a crucial role in the maintenance of pregnancy from conception to about 100–120 days of gestation when placenta becomes the main source of gestagens. Primary corpus luteum is the sole source of progesterone until the time when secondary or accessory corpora lutea are formed, which occurs closely after appearance of endometrial cups, and manifests with an increase in progesterone concentration in the systemic circulation [4]. During the first five weeks of pregnancy, when primary corpus luteum is the only source of progesterone, the highest incidence of EED is recorded; however, there is no clear evidence that dysfunction of the primary corpus luteum can indeed cause pregnancy loss [1, 4].

During the last few breeding seasons, we frequently observed that, while the development of ovarian follicles was normal (i.e., they reached a minimum diameter of 4 cm) and ovulation occurred, endometrial edema not always fully developed during estrus. In some mares, the maximum endometrial edema noted during estrus was grade 1 according to the system proposed by Samper [6], which can suggest poor estrogenization. Interestingly, it was shown that the duration of estrus-like echotexture shorter than 4 days, both in induced and spontaneous estrus is associated with reduced pregnancy rate [7]. Our hypothesis was that the low endometrial edema during estrus may influence the future function of the corpus luteum.

## Results

Progesterone concentration at 14 days post-ovulation was significantly higher in group A than group B mares, with no significant differences detected for any of the other parameters evaluated (Table 1).

**Table 1:** Comparisons of evaluated parameters between group A and B

	Age of the mare	Endometrial edema	Depth of intrauterine fluid	Number of uterine flushings	Time from AI to the day when uterus was found free of fluid	Progesterone concentration (ng/ml)
Group A	10.8 ± 4.5	1.8 ± 0.7	1.9 ± 1.4	0.8 ± 0.7	2.5 ± 1.8	11.32 ± 5.26^a^
Group B	9.4 ± 4.0	1.4 ± 0.6	1.2 ± 1.2	0.5 ± 0.7	2.4 ± 2.0	4.23 ± 1.20^a^

Values described with letter a—significant difference at *P* < 0.001

Fourteen days after ovulation an increase in endometrial edema was never seen. In group A, progesterone concentrations measured at 14 days after ovulation ranged from 6.27 to 19.69 ng/ml. In group B, enrolling mares with a potential diagnosis of primary corpus luteum insufficiency, progesterone concentrationss at this time point ranged from 1.15 to 6.07 ng/ml. In this latter group, the concentration of progesterone was below 2 ng/ml in two mares, equal to 2.62 ng/ml in one mare, within the 3–4 ng/ml range in four mares, and above 4 ng/ml in the remaining 15 mares.

Correlation coefficients between progesterone concentration and other assessed parameters for mares in groups A and B are showed in Table 2 and 3, respectively.

**Table 2:** Correlations between progesterone concentration in the blood and other investigated parameters in the group A

Age of the mare	Endometrial edema	Depth of intrauterine fluid	Number of uterine flushings	Time from AI to the day when uterus was found free of fluid
*r*_s_ = -0.563	*r*_s_ = 0.299	*r*_s_ = 0.137	*r*_s_ = 0.128	*r*_s_ = 0.090
*P* = 0.064	*P* = 0.321	*P* = 0.656	*P* = 0.676	*P* = 0.770

**Table 3:** Correlations between progesterone concentration in the blood and other investigated parameters in mares with small CL or with small embryonic vesicle (group B)

Age of the mare	Endometrial edema	Depth of intrauterine fluid	Number of uterine flushings	Time from AI to the day when uterus was found free of fluid
*r*_s_ = 0.233	*r*_s_ = 0.753	*r*_s_ = 0.184	*r*_s_ = 0.108	*r*_s_ = 0.338
*P* = 0.297	*P* < 0.001	*P* = 0.412	*P* = 0.632	*P* = 0.124

A significant correlation was found only between progesterone concentration measured 14 days after ovulation and endometrial edema evaluated during estrus in mares from group B. In this group, 15 mares had a maximum edema score of 1, six mares had a maximum edema score of 2, and one mare had a maximum edema score of 3.

Course of pregnancy in mares from group A was always normal. Fourteen mares from group B were treated with altrenogest due to low progesterone concentration. In five of these mares, despite of altrenogest treatment EED occurred. In one mare (not treated with altrenogest) from group B loss of pregnancy occurred at 60 days after ovulation, and this could be connected with colic signs occurred 7 days earlier. The rest of pregnancies in this group had normal course.

## Discussion

Diseases of the endometrium are widely considered one of the most important causes of EED [1, 8, 9]. Endometritis leads to the release of prostaglandin F_2_α, which may cause low progesterone production. Accumulation of IUF after natural breeding or AI is associated with a reduction in pregnancy rates and favors the occurrence of EED [9]. In our study, we only enrolled mares that were pregnant 14 days after AI, and therefore could not evaluate the impact of endometritis on EED. Nevertheless, our results suggest that the presence of IUF, the number of required treatments against IUF, and the time from AI to the day when the uterus was found free of fluid had no impact on progesterone concentration measured 14 days after ovulation. However, none of the mares in our study retained fluid longer than 5 days from ovulation. We also did not find correlations between the age of the mares and progesterone concentration measured 14 days after ovulation.

It should be mentioned that our study has some limitations. First of all, the material used in our study was collected in the field conditions from mares introduced into a commercial AI program, and it was not possible to create perfectly homogeneous groups of animals. Additionally, all results were analysed retrospectively, and hormone concentrations were not measured during estrus and we cannot explain the reasons for the lack of normal estrus edema. Consequently, our work should be considered as preliminary research, and further studies are necessary for better understanding the reasons, and role of poor endometrial edema on outcomes of mares fertility.

Despite of altrenogest treatment EED occurred in some mares from group B. This support thesis that reasons leading to EED are multifactorial. Although luteal deficiency is usually at the end of the list of probable causes of EED, supplementation with altrenogest is more and more common. Very often it is used more as a precaution than based on medical grounds. Generally, progesterone concentration below 2 ng/ml in the blood is associated with embryonic loss, whereas concentration above 4 ng/ml is considered to be sufficient for embryo survival [10]. In our study, only two mares had progesterone concentrations lower than 2 ng/ml and in five others, the concentrations ranged from 2 to 4 ng/ml. The other 15 mares from group B had progesterone concentrations above 4 ng/ml, but still close to this level (range: 4.12 to 6.07 ng/ml). In contrast, progesterone concentrations in group A could reach up to 19 ng/ml and a significant difference was found in progesterone concentrations between mares with small (group B) and normal (group A) corpora lutea.

It has also been noted that progesterone insufficiency can lead to embryonic developmental disorders [11], and that smaller embryonic vesicle size noted at days 13–15 of pregnancy may predispose to EED [12]. In our study, only one embryonic vesicle had a diameter < 14 mm at 14 days after ovulation, corresponding to a progesterone concertation of 3.33 ng/ml. The corpus luteum in this mare had a diameter of more than 15 mm, and the endometrial edema pattern was typical for normal early pregnancy (i.e., no increase in edema was noted). However, the examinations performed daily do not allow to identify exactly the moment of ovulation. Ovulation induced by hCG may occur between 30–42 h after administration. Thus, it cannot be excluded that this embryonic vesicle was normal. According to Newcombe [12], a pathological increase in endometrial edema can sometimes be noticed in early pregnancy, even in the presence of a normal embryonic vesicle, and this abnormal edema coincides with progesterone concentrations of less than 1 ng/ml. In our study, we identified no cases of an increase in endometrial edema at 14 days after ovulation, which can be explained by the fact that progesterone concentration was always above 1 ng/ml.

We found a significant correlation between progesterone concentration measured 14 days after ovulation and endometrial edema evaluated during estrus in mares with a small corpus luteum. In contrast, the same correlation was not found in mares with a normal corpus luteum. It was shown that opposite situation i.e. increased endometrial edema observed during estrus could indicate uterine pathology [6, 13]. Increasing endometrial edema measured early in the estrus is a risk factor for subclinical persistent infection with β-hemolytic streptococci [13].

During typical estrus, endometrial edema slowly increases and then begins decreasing when ovulation approaches [6, 14]. Reduction of pregnancy rate was observed in mares with absence of estrus-like echotexture during estrus [7]. Additionally, in the same study was shown that the duration of endometrial edema phase influenced the pregnancy rate in mares treated with PGF_2_α or with spontaneous estrus [7]. If endometrial edema score increases 8 h after mating, regardless of the initial score at the time of mating, a reduced conception rate can be expected [15]. In mares included in this study, endometrial edema often did not exceed a score of 1. Edema pattern of the uterus depends on an interaction between estrogens and progesterone [16]. However, in the study conducted by Pycock et al. [17], no direct correlation was found between endometrial edema and estradiol concentrations. As we did not assess estrogen concentrations, we could not ascertain if the low endometrial edema observed in some mares in our study was caused by low production of estrogens. Low endometrial edema observed in our study cannot be explain by incomplete luteolysis because all mares were frequently and carefully examined ultrasonographically. We cannot explain the reasons for low endometrial edema.

Induction of ovulation using hCG results in cessation of growth of the dominant follicle and is associated with decreasing scores for endometrial echotexture and a fall in the blood estradiol concentration [18]. Generally, the concentrations of estrogens decrease as ovulation approach [18]. It has been demonstrated that manipulation of the estrous cycle through administration of cloprostenol or induction of ovulation with hCG or deslorelin can result in increased uterine edema scores in the periovulatory period compared with non-induced cycles; however, this tendency was only significant for the treatment with cloprostenol [14]. In our study, all mares were treated with hCG but endometrial edema score was recorded at the highest point prior to hCG administration, so that no effect of the ovulation-inducing agent on edema was captured.

Based on published data, it seems that primary corpus luteum dysfunction cannot be a common cause of EED [8, 19]. However, in some mares with EED, a decrease in progesterone concentration has been observed from 96 h after ovulation [20]. Betteridge et al. [21] described eleven cases of spontaneous pregnancy loss which occurred between gestation days 13 and 25, among which 6/11 losses were associated with luteolysis while 5/11 were not. Furthermore, Beyer et al. [22] found no differences in the diameter of embryonic vesicles (measured from 10 to 14 days post ovulation) in healthy mares treated with altrenogest at days 5–10 after ovulation, with cloprostenol at days 0–3 days after ovulation, and in untreated mares. These results can indicate that lower progestin concentrations during early pregnancy do not influence the development of the early conceptus. However, another study has shown that 8 day old embryos collected from mares treated with dinoprost to reach progesterone concentration less than 1 ng/ml displayed increased expression of four transcripts related to steroid hormone signaling, embryonic capsule formation, and nutrient transport compared to embryos collected from a uterine environment influenced by a typical early pregnancy progesterone concentration [23]. Additionally, 8-day old embryos collected from a progesterone-deprived environment have been reported to be smaller and of lower quality than embryos collected from unaffected mares [24]. Thus, adequate progesterone concentration during early pregnancy can influence both gene expression and the size of embryos [23, 24]. In addition, a study conducted by Okada et al. [11] showed that sub-physiological progestin concentrations in the early postovulatory period had a negative impact on pregnancy development before placentation in horses. The newest observations support, at least in part, the notion that luteolysis may play a bigger role in the pathogenesis of EED than commonly described [21].

Allen [4] suggested that widespread supplementation of pregnant mares with exogenous gestagens is questionable and that there is no evidence that routine administration of progesterone/gestagens is rational even in mares with a history of EED. However, it has been shown that supplementation with altrenogest from 6 days post-ovulation had no adverse impact on pregnancy rate and, in fact, positively influenced conceptus development in older mares [25]. Thus, administration of altrenogest in early pregnancy may have a positive effect on the course of pregnancy.

## Conclusion

In some cases poor development of endometrial edema during estrus can be associated with lower progesterone production 14 days after ovulation. Nevertheless, scientific explanation for this finding cannot be given based on our study.

## Methods

### Study aim

The aim of the study was to test progesterone concentration 14 days after ovulation in pregnant mares and relate it to peak estral endometrial edema and the presence of intrauterine fluid (IUF) after artificial insemination (AI), the number of treatments against IUF, and the time from AI to the day when the uterus was found free of fluid.

### Animals and experimental groups

From a number of mares introduced into a commercial AI program, 35 warmblood mares were enrolled for the study. Their age ranged from 3 to 18 years, and body weight from 500 to 650 kg. All mares had a good body condition score (average 6/9). The animals were stabled overnight and turned out during daytime for a minimum of 6 h per day, providing exposure to natural light. Their diet consisted of grass hay fed ad libitum and 2 kg of concentrate per day.

Based on the features of the early pregnancy, the mares were divided into two groups (A – normal and B – potential primary corpus luteum insufficiency). In group A (*n* = 13; age 10.8 ± 4.5 years), a normal embryonic vesicle with a diameter ≥ 14 mm and a corpus luteum with a diameter ≥ 15 mm were found 14 days after ovulation. In group B (*n* = 22; age 9.4 ± 4.0 years), a small (< 15 mm) corpus luteum and/or a small embryonic vesicle were observed (diameter < 14 mm) at 14 days after ovulation. In the group A, five mares were barren, 2 mares were with foal by side and 6 mares were maiden. In the group B, three mares were barren, 13 with foal by side, and 6 mares were maiden. All barren mares from group A and B had a history of unexplained EED. In the previous breeding season, material for cytological and microbiological examinations were collected from the uterus using double-guarded swab (Equi-Vet, Kruuse, Denmark) from all barren mares, and results of these examinations showed no infection, and no inflammation of the endometrium. Only mares with a single ovulation were included in the study, and all mares were clinically healthy throughout the duration of the study.

### Examination of the reproductive tract, AI, and treatment for uterine fluid

All mares were examined by transrectal palpation and ultrasonography using a Draminski Blue ultrasound machine equipped with a 5–10 MHz multifrequency linear rectal transducer. Six mares from group A, and 7 mares from group B were treated *i.m.* with 5 mg of dinoprost (Dinolytic®, Zoetis, Poland) for estrus induction. Three mares from group B ovulated by 7 days after treatment. Mares were checked daily during estrus for genital health and detection of optimal time for induction of ovulation prior to AI. All mares included in this study had no fluid in the uterus during estrus, endometrial edema was no excessive, and conformation of the perineal area was normal. When a dominant follicle with a diameter of ≥ 4 cm was detected, the mares were treated *i.v.* with 1500 IU of human chorionic gonadotrophin (hCG) (Chorulon®, Intervet, Holland) and inseminated approximately 24 h after the induction of ovulation with cooled semen obtained from stallions with proven fertility.

**Fig. 1:**
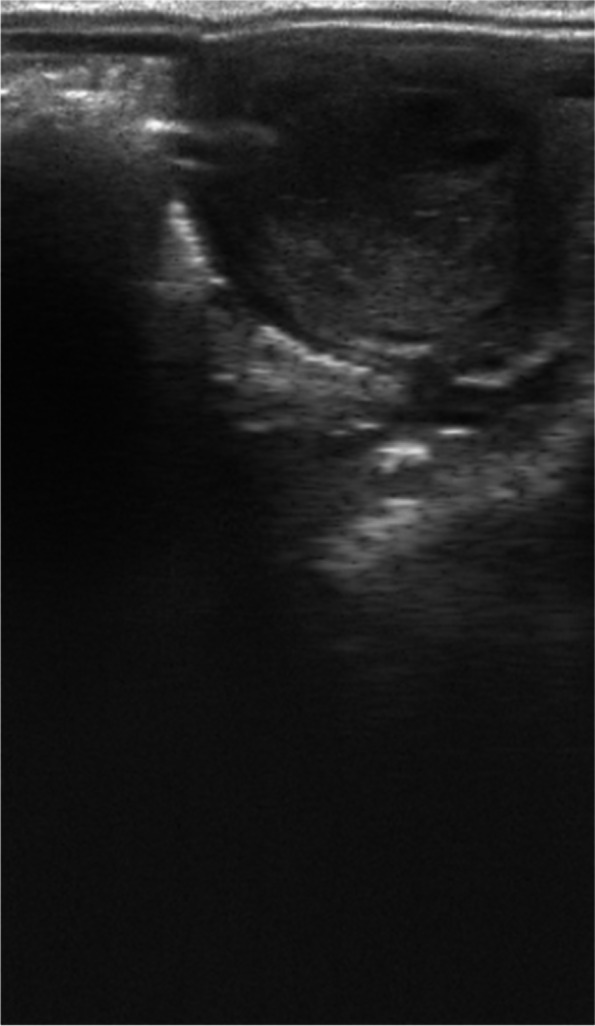
No endometrial edema, score 0

**Fig. 2:**
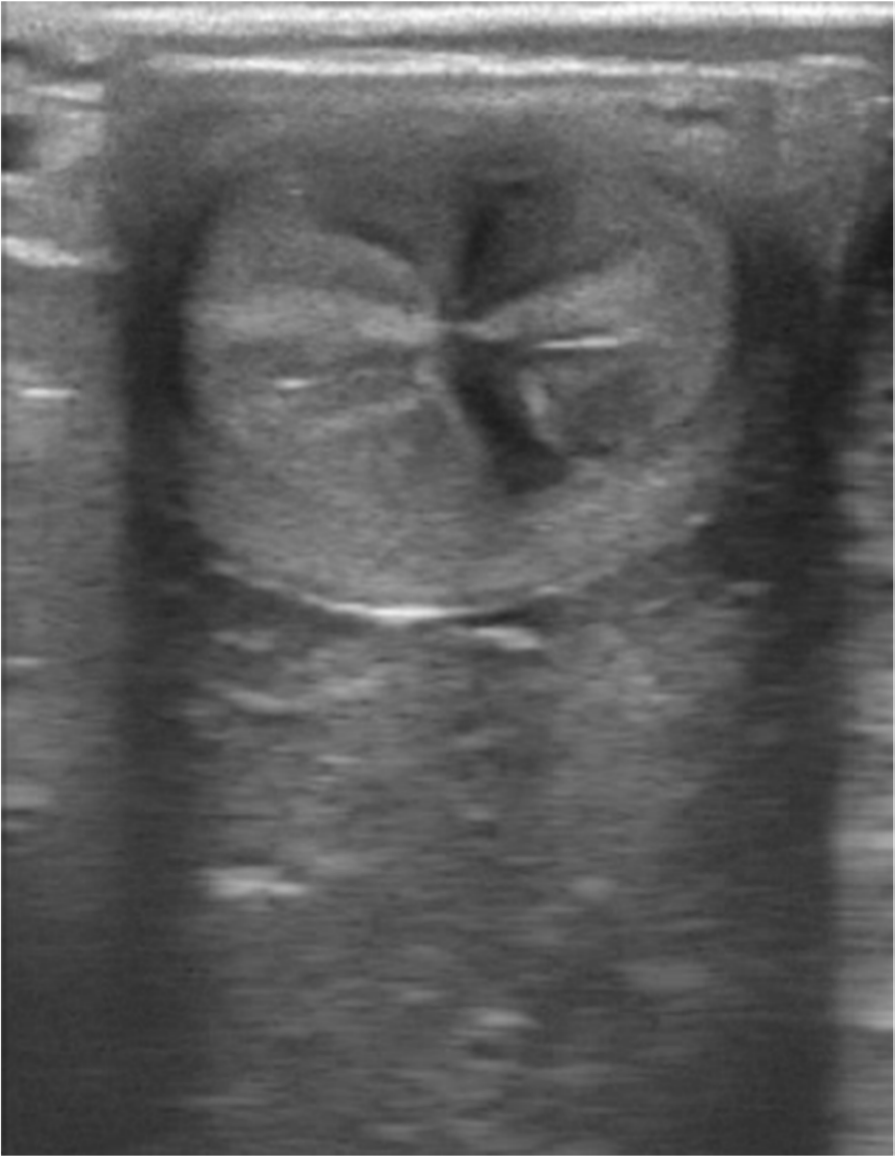
Mild edema, score 1

**Fig. 3:**
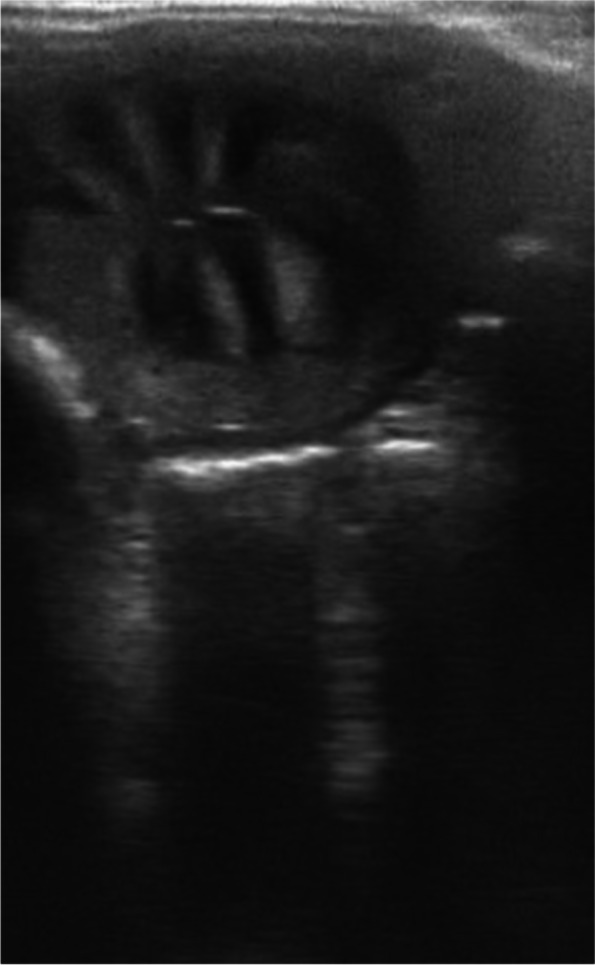
Moderate edema, score 2

**Fig. 4:**
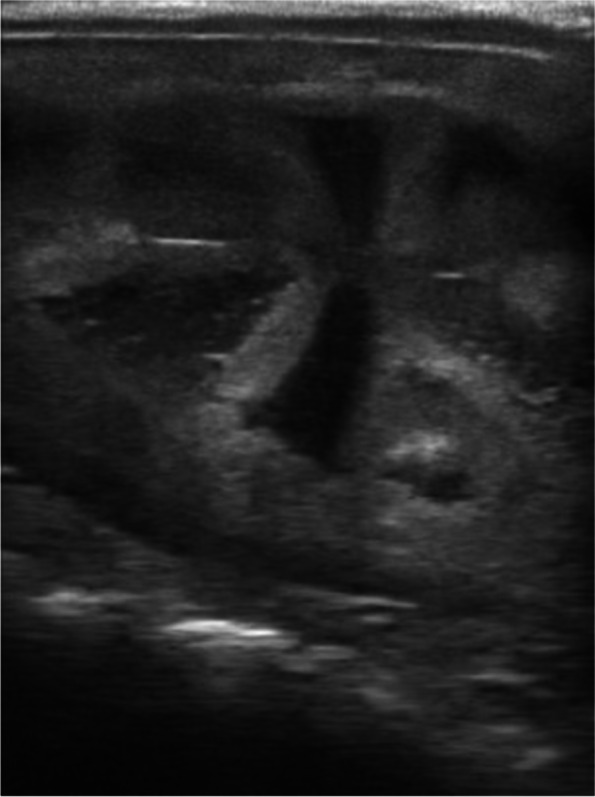
Strong edema, score 3

During estrus endometrial oedema was scored according to the system proposed by Samper [6], and modified by Rasmussen et al. [15] whereby a score of 0 indicates no edema and a score of 5 corresponds to abnormal hyper-edema. The following figures show the adopted criteria for evaluation of endometrial edema: Fig. 1. No endometrial edema, score 0; Fig. 2. Mild edema, score 1; Fig. 3. Moderate edema, score 2. Figure 4 Strong edema, score 3. The highest endometrial edema score observed during estrus was included in the analysis.

Before AI, the tail was protected using an examination glove and the perineum and vulva were washed using soap and water, and subsequently scrubbed with povidone-iodine and dried with a paper towel. Semen was deposited into the uterine body using a sterile catheter with the operator’s hand covered with a sterile glove and inserted into the vagina.

Ultrasound examination of the reproductive tract was also performed daily after AI, to detect ovulation and check for the presence of IUF. The depth of IUF measured 24 h after AI was included in the analysis and was scored as follows: score 0 – no fluid; score 1 – a line of fluid; score 2 – depth up to 10 mm; score 3 – depth from 11 up to 20 mm; and score 4 – depth ≥ 20 mm. If IUF was found on ultrasound examination, the mare was prepared for flushing in the same way as for AI and the uterus was flushed with warm saline solution (1-3L per treatment, depending on the case) using a sterile flushing tube (EQUIVET Uterine Flushing, Kruuse, Denmark). Additionally, 20 IU of oxytocin were given i.m. 3 times every 4 h apart. The ultrasound examination and flushing were repeated daily until no more IUF was detected. The number of treatments and the day after AI when no fluid was detected in the uterus were recorded.

All mares enrolled into the study ovulated within 24 h after semen deposition. Fourteen days after ovulation, an ultrasound examination was performed for pregnancy diagnosis and evaluation of the corpus luteum. Additionally, endometrial oedema was evaluated. The diameter of the corpus luteum was obtained as the average of transverse and longitudinal measurements. Following the examination, blood from the external jugular vein was collected into 10 ml tubes for determination of progesterone concentration.

### Determination of progesterone concentration

After collection, blood was allowed to clot at room temperature and the samples were centrifuged at 1500 g for 15 min to obtain serum. Serum samples were stored and transported to the commercial laboratory at 4 °C. Progesterone concentration was determined by a commercial automated immunoassay (Immulite® 2000 progesterone, Siemens Healthcare Diagnostics Products Ltd., UK). Immulite 2000 progesterone is a solid-phase, competitive chemiluminescent enzyme immunoassay with an analytical sensitivity of 0.1 ng/ml and a calibration range of 0.2–40 ng/mL. The assay has been previously used for the determination of pregnanes, including progesterone, in mares [26, 27] and has demonstrated a strong correlation with testing utilizing the radioimmunoassay technique [26].

### Statistical analysis

The relationship between progesterone concentration and: a) the age of the mare; b) the score of endometrial edema; c) the depth of intrauterine fluid; d) the number of uterine flushings and e) the time from AI to the day when uterus was found free of fluid were calculated separately for the group A (*n* = 13), where a normal embryonic vesicle with a diameter ≥ 14 mm and a corpus luteum with a diameter ≥ 15 mm were found 14 days after ovulation, and for group B (*n* = 22), where a small (< 15 mm) corpus luteum and/or a small embryonic vesicle were observed (diameter < 14 mm) at 14 days after ovulation. Due to the ordinal scale of the variables or the lack of normality of the distribution, the determination of the dependence was based on the calculation of the Spearman's rank correlation coefficient (r_s_), and then its statistical significance test was performed.

Then, a separate analysis of the mean values ​​of the variables: a) the age of the mare; b) the score of endometrial edema; c) the depth of intrauterine fluid; d) the number of uterine flushings and e) the time from AI to the day when uterus was found free of fluid were calculated using Mann–Whitney U test for comparisons between groups A and B. All statistical analyses were performed using STATISTICA version 13 (TIBCO Software Inc. 2017).

## Data Availability

The datasets used and analysed during the current study are available from the corresponding author on reasonable request.

## Abbreviations

IUF: Intrauterine fluid
AI: Artificial insemination
EED: Early embryonic death
